# The Value of the History and Physical for Patients with Newly Diagnosed Brain Metastases Considering Radiosurgery

**DOI:** 10.3389/fonc.2016.00040

**Published:** 2016-03-02

**Authors:** Ima Paydar, Eric Karl Oermann, Miriam Knoll, James Lee, Brian Timothy Collins, Matthew Ewend, Douglas Kondziolka, Sean P. Collins

**Affiliations:** ^1^Department of Radiation Medicine, Georgetown University Medical Center, Washington, DC, USA; ^2^Department of Neurological Surgery, Mount Sinai Health System, New York, NY, USA; ^3^Department of Radiation Oncology, Mount Sinai Health System, New York, NY, USA; ^4^Department of Neurological Surgery, The University of North Carolina at Chapel Hill, Chapel Hill, NC, USA; ^5^Department of Neurosurgery, New York University Langone Medical Center, New York, NY, USA

**Keywords:** history and physical, radiosurgery, brain metastases, performance status, neurological deficits, prognosis, comorbidity

## Abstract

**Background:**

For patients with brain metastases, systemic disease burden has historically been accepted as a major determinant of overall survival (OS). However, less research has focused on specific history and physical findings made by clinicians and how such findings pertain to patient outcomes at a given time point. The aim of this study is to determine how the initial clinical assessment of patients with brain metastases, as part of the history and physical at the time of consultation, correlates to patient prognosis.

**Methods:**

We evaluated a prospective, multi-institutional database of 1523 brain metastases in 507 patients who were treated with radiosurgery (Gamma Knife or CyberKnife) from 2001 to 2014. Relevant history of present illness (HPI) and past medical history (PMH) variables included comorbidities, Eastern Cooperative Oncology Group (ECOG) performance status, and seizure history. Physical exam findings included a sensory exam, motor exam, and cognitive function. Univariate and multivariate Cox regression analyses were used to identify predictors of OS.

**Results:**

Two hundred ninety-four patients were included in the final analysis with a median OS of 10.8 months (95% CI, 7.8–13.7 months). On univariate analysis, significant HPI predictors of OS included age, primary diagnosis, performance status, extracranial metastases, systemic disease status, and history of surgery. Significant predictors of OS from the PMH included cardiac, vascular, and infectious comorbidities. On a physical exam, findings consistent with cognitive deficits were predictive of worse OS. However, motor deficits or changes in vision were not predictive of worse OS. In the multivariate Cox regression analysis, predictors of worse OS were primary diagnosis (*p* = 0.002), ECOG performance status (OR 1.73, *p* < 0.001), and presence of extracranial metastases (OR 1.22, *p* = 0.009).

**Conclusion:**

Neurological deficits and systemic comorbidities noted at presentation are not associated with worse overall prognosis for patients with brain metastases undergoing radiosurgery. When encountering new patients with brain metastases, the most informative patient-related characteristics that determine prognosis remain performance status, primary diagnosis, and extent of extracranial disease.

## Introduction

For patients with brain metastases undergoing radiation treatment, patient-specific variables such as performance status and extracranial disease burden are commonly used to prognosticate overall survival (OS). Numerous therapies exist to treat such systemic and intracranial disease, including chemotherapy, radiation, and supportive care; therefore, patient selection must be a priority before initiating treatment because each therapy has unique risks and benefits. Several prognostic indices have been developed for patients with brain metastases to improve patient selection for and to predict outcomes after treatment.

In the seminal predictive models of patient outcomes, baseline neurological function at presentation has inconsistently been included as a potential prognostic variable. In an analysis of patients included in three Radiation Therapy Oncology Group (RTOG) studies undergoing whole brain fractionated radiotherapy, neurological function was included as a potential prognostic factor in the regression analysis (1). Although neurological function was not one of the variables that ultimately defined prognostic groups on recursive partitioning analysis (RPA), it was a statistically significant prognostic variable on univariate analysis. Importantly, the RTOG RPA, which enrolled patients as early as 1979, did not include patients treated with radiosurgery, and, therefore, its results may not be generalizable to patients with intracranial metastases treated with radiosurgery alone. Other prognostic indices published in the more modern era were based on analyses of patients treated with either whole brain radiation therapy (WBRT) (2, 3), stereotactic radiosurgery (4, 5), or either one of these modalities (6–8). These indices did not evaluate neurological function as a potential prognostic indicator; hence, it was not included in the resulting scoring systems.

Most key prognostic indices include a measure of performance status, such as Karnofsky performance status (KPS) score or Eastern Cooperative Oncology Group (ECOG) score. These scores are determined by the examining clinician, attempt to quantify the general well-being of patients, and are often independent of the underlying pathology. A recent study reported that a neurological cause of a low KPS score was associated with a statistically significant improved prognosis in patients who undergo radiosurgery (9). Moreover, another published nomogram provides individual estimates of neurological death after salvage stereotactic radiosurgery for patients who have failed prior WBRT after adjusting for their competing risk of death from non-neurological causes (10). These findings underscore the potential prognostic value of neurological deficits elicited on a physical exam in determining outcomes for patients with brain metastases who undergo treatment with radiosurgery.

The goal of our study was to determine whether neurological deficits noted prior to radiation treatment predicted for worse outcomes in patients treated with stereotactic radiosurgery for brain metastases. To the best of our knowledge, this is the only multi-institutional review examining the relevance of the neurological examination when determining a patient’s prognosis in the setting of radiosurgery. Other patient-related and treatment-related variables were also assessed to delineate potential prognostic variables from this patient population.

## Materials and Methods

### Participant Selection and Study Design

**Figure 1:**
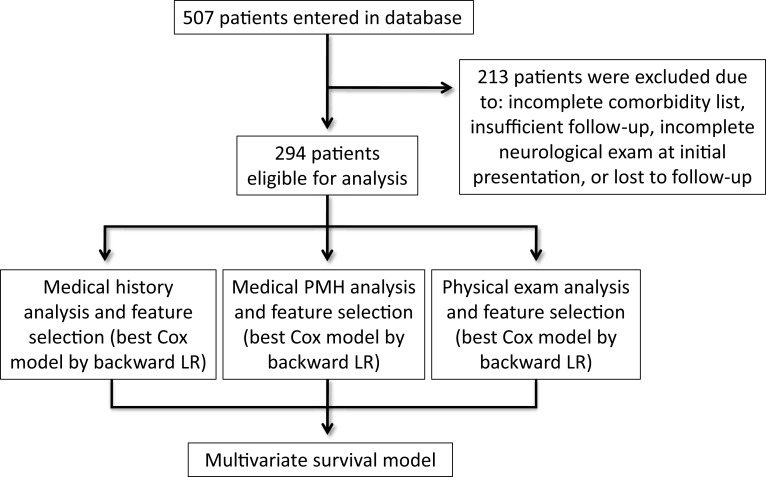
**Flow chart representing the patient selection process and analytic plan**.

We performed a retrospective evaluation of a prospectively collected, multicenter database of 1523 brain metastases in 507 patients who were treated with radiosurgery (Gamma Knife or CyberKnife) between 2001 and 2014. Institutional Review Board approval for the accumulation of clinical data for the purpose of this study was obtained from The University of North Carolina at Chapel Hill, New York University, and Georgetown University Hospital. From the 507 patients, 213 patients were excluded because they had received prior surgery or intracranial radiation or had been lost to follow-up. The remaining 294 patients with newly diagnosed brain metastases, biopsy-confirmed primary or metastatic disease, and no prior treatment with intracranial radiation were included in the final analysis (Figure 1).

### Baseline Variables and Treatment

The patient variables included gender, age, primary diagnosis/histology, total intracranial tumor volume, systemic disease burden, and systemic disease control. Systemic disease burden was determined by positron emission tomography (PET)/CT and was quantified by the number of major organ systems with active metastatic lesion. Systemic disease control was defined as improved or stable disease over serial PET/CT. Radiosurgical treatment planning was carried out as previously described (11), and final fractionation and dosage was determined based on the gross tumor volume (GTV) and proximity to critical structures. Patients could receive further treatment including WBRT, surgical resection, or repeat radiosurgery if they failed initial therapy or developed symptomatic mass effect. These treatments were administered at the discretion of the treating physicians.

### Outcomes Assessment

Radiographic control of treated intracranial lesions was assessed by serial magnetic resonance imaging (MRI) obtained as a part of routine clinical follow-up. Patients were imaged every 2–3 months after radiosurgery at the discretion of the treating radiation oncologist and neurosurgeon. For patients with multiple intracranial lesions at the time of radiosurgery or who developed new lesions distal to the site of treatment, local control (LC) was defined as control of the specific lesions treated with radiosurgery. OS was calculated from the last day of treatment until the date of death. All patients were monitored clinically for radiosurgery-associated toxicity throughout the length of follow-up, and all such toxicities observed were recorded and scored utilizing the Common Toxicity Criteria for Adverse Events version 4.0 (CTCAE v.4).

### Statistical Analysis

Between-group differences were assessed using the log-rank test or Chi-square test within each of the study cohorts for categorical data. Ordinal variables were assessed utilizing gamma (a type of rank correlation). Cumulative event rates for individual variables were calculated according to the Kaplan–Meier method with differences in survival curves assessed using the log-rank method. Multivariate Cox proportional-hazards regression models were constructed for the overall study population using variables found to be significant (α = 0.05) on univariate analysis. All reported ranges are interquartile ranges (IQR) defined as the range between the 25th percentile and the 75th percentile. All reported *p* values are two sided, with an alpha of 0.05. All data management and analyses were conducted using SPSS 21.0 (IBM, Inc., Armonk, NY, USA) as well as the open source SCikit-learn library in Python.

## Results

### Patient and Treatment Characteristics

Two hundred ninety-four patients with brain metastases were included in the final analysis. The median age at diagnosis was 63, and 57% were male. The most common primary diagnosis was non-small cell lung cancer (NSCLC) (52%). The majority of treated patients (83%) had an ECOG performance status of 0 or 1 and low extra-cranial disease burden (63% with 0–1 sites of disease). However, extracranial disease was uncontrolled in the majority of patients (67%). The median number of intracranial metastases was 2, and the median tumor volume was 1.7 mm^3^. Thirty-six percent of patients presented with a neurological complaint, the most common of which was a motor deficit (15%). For the remainder of the patients, brain metastases were noted on routine surveillance imaging (64%). Patients were treated with a median dose of 19 Gy (IQR, 18–20 Gy) in one (91%), two (8%), or three (1%) fractions. Specifics of baseline patient characteristics are shown in Tables 1 and 2.

**Table 1:** **Baseline characteristics of patients and treatments with newly diagnosed brain metastases**.

Patient characteristic	
Gender – no. (%)	
Male	127 (57)
Female	167 (43)
Age – years (IQR)	63 (55–70)
Total intracranial tumor volume – mm^3^ (IQR)	1.7 (0.7–3.7)
Primary diagnosis – no. (%)	
NSCLC	152 (52)
Breast cancer – all types	63 (22)
Melanoma	64 (22)
Renal cell carcinoma	15 (5)
ECOG – no. (%)	
0	93 (32)
1	151 (51)
2	39 (13)
3+	11 (4)
Extracranial metastatic burden – no. sites (%)	
0	63 (21)
1	124 (42)
2	52 (18)
3	27 (9)
4	21 (7)
5+	7 (2)
Systemic disease status – no. (%)	
Controlled	96 (33)
Uncontrolled	198 (67)
Number of metastases – median no. (IQR)	2 (1–4)
Whole brain radiation therapy – no. (%)^a^	52 (23)
Cranial surgery – no. (%)^a^	62 (21)
Isodose % (IQR)	50 (50–80)
Isocenters – median (IQR)	3 (1–6)
Dose – median (IQR)	19 (18–20)
Fractions – no. (%)	
1	267 (91)
2	25 (8)
3	2 (1)

*^a^Treatment received after SRS*.

**Table 2:** **Baseline neurological examination and symptoms**.

Patient characteristic	
Seizures – no. (%)	21 (7)
Motor deficits – no. (%)	45 (15)
Cognitive deficits – no. (%)	28 (10)
Vision loss – no. (%)	13 (4)

### Overall Survival and Local Control

**Figure 2:**
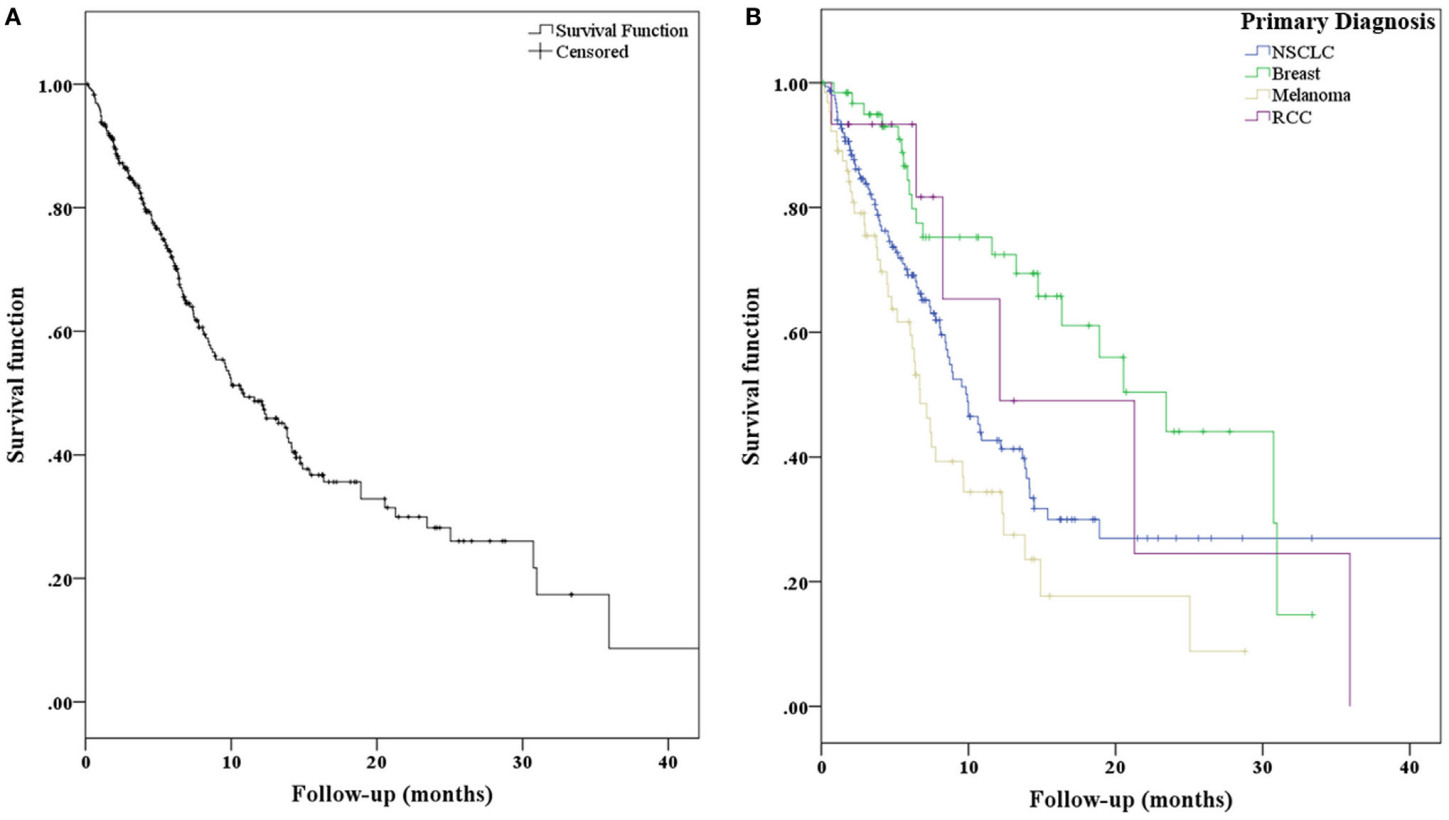
**(A)** Kaplan–Meier curves representing overall survival for all patients and **(B)** separated with respect to primary diagnosis.

For the entire patient population, the median OS was 10.8 months (95% CI, 7.8–13.7 months). With respect to primary diagnosis, patients with breast cancer demonstrated the best median OS (23.4 months; 95% CI, 15.7–31.2 months), followed by renal cell carcinoma (12.1 months; 95% CI, 0.1–24.2 months), NSCLC (9.9 months; 95% CI, 8.1–11.7 months), and melanoma (6.7 months; 95% CI, 5.4–8.0 months) (Figures 2A,B). Freedom from local recurrence was 94% over the course of the study with a median freedom from local recurrence of 41 months (95% CI, 37.6–44.7 months).

### Modeling Initial Presentation Survival Predictors

**Figure 3:**
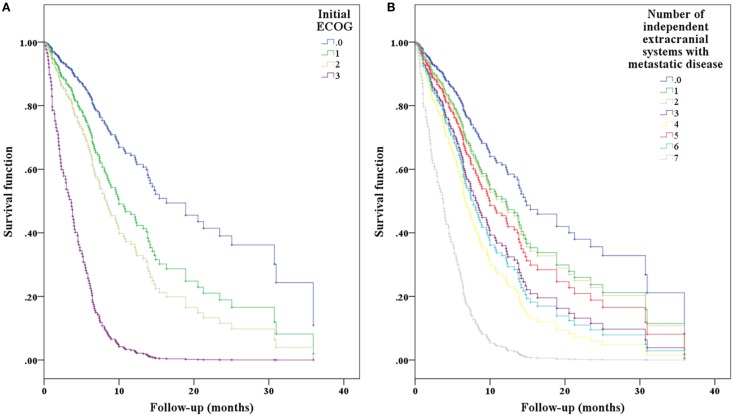
**Cox-hazard regression results separated by (A) ECOG performance status and (B) extracranial disease status demonstrating the expected effect of worsening performance status and systemic disease on overall predicted survival**.

In the univariate Cox regression analysis, key predictors of worse OS from the history of present illness (HPI), past medical history (PMH), and neurological exam were older age (OR 1.03, *p* = 0.001) and site of primary disease (*p* < 0.001) (Table 3). Specifically, compared to NSCLC, melanoma had a slightly increased risk (OR 1.63, *p* = 0.022), while breast carcinoma (OR 0.62, *p* = 0.084) and RCC (OR 0.45, *p* = 0.077) had decreased risk. Other predictors of worse OS were ECOG performance status (OR 1.55, *p* < 0.001); presence of extracranial metastases (OR 1.20, *p* = 0.005); systemic disease burden (OR 2.36, *p* < 0.001); a PMH of cardiac disease (OR 2.06, *p* = 0.005), vascular disease (OR 1.87, *p* = 0.004), or infectious disease (OR 3.53, *p* = 0.042); and a neurological exam demonstrating cognitive decline (OR 2.03, *p* = 0.007). The presence of any neurological deficit was also a statistically significant predictor of worse OS on univariate analysis (OR 1.48, *p* = 0.043). Predictors of improved OS were presence of intracranial metastases only (OR 0.89, *p* = 0.526) and history of cranial surgery after radiosurgery (OR 0.59, *p* = 0.025). In the multivariate Cox regression analysis, predictors of worse OS were primary diagnosis (*p* = 0.002), ECOG performance status (OR 1.73, *p* < 0.001), and presence of extracranial metastases (OR 1.22, *p* = 0.009) (Figures 3A,B; Table 4). Neurological deficits assessed individually or all together were not statistically significant predictors of OS (OR = 1.32 [0.84–2.06], *p* = 0.23 for deficits assessed together).

**Table 3:** **Predictors of overall survival from HPI, PMH, and neurological exam on univariate analysis**.

Patient characteristic	OR (95% CI)	*p*-Value
**History of present illness**		
Seizures	1.57 (0.82–3.0)	0.170
Age	1.03 (1.01–1.04)	0.001
Primary diagnosis^a^	–	<0.001
Breast carcinoma	0.62 (0.36–1.07)	0.084
Melanoma	1.63 (1.07–2.46)	0.022
RCC	0.45 (0.19–1.09)	0.077
ECOG	1.55 (1.24–1.94)	<0.001
Extracranial metastases^b^	1.20 (1.06–1.37)	0.005
Intracranial metastases^c^	0.89 (0.63–1.27)	0.526
Systemic disease status	2.36 (1.59–3.52)	<0.001
History of cranial surgery	0.59 (0.37–0.94)	0.025
History of WBRT	0.86 (0.58–1.24)	0.425
**Past medical history**		
Cardiac	2.06 (1.25–3.41)	0.005
Vascular	1.87 (1.23–2.83)	0.004
Infectious	3.53 (1.05–11.9)	0.042
**Neurological exam**		
Motor deficit	1.12 (0.70–2.01)	0.515
Vision loss	1.36 (0.60–3.10)	0.459
Any neurological deficit	1.48 (1.01–2.15)	0.043

*^a^Odds ratio is compared to a baseline of NSCLC*.

*^b^OR is for every additional independent system with active metastatic disease*.

*^c^Median number for series was two metastases, treated as over/under two metastases or oligometastases versus multiple metastases*.

**Table 4:** **Multivariate model of overall survival**.

Patient characteristic	OR (95% CI)	*p*-Value
History of present illness		
Primary diagnosis^a^	–	0.002
ECOG	1.73 (1.34–2.21)	<0.001
Extracranial disease burden^b^	1.22 (1.05–1.42)	0.009
Past Medical History – N/A		
Neurologic exam – N/A		

*^a^Odds ratio varies depending upon specific diagnosis/histology with Melanoma > NSCLC > RCC > Breast*.

*^b^OR is for every additional independent system with active metastatic disease*.

### Toxicity

All patients were able to complete radiation treatment without interruption. Patients were also able to tolerate treatment with minimal toxicity. Specifically, 5% of patients experienced acute grade 1–2 toxicity. Two patients (0.7%) experienced intratumoral hemorrhages post-radiosurgery, and two patients (0.7%) had new seizures. The most common treatment-related side effects were headaches, nausea, and alopecia; these were medically managed, often with corticosteroid therapy.

## Discussion

Brain metastases are the most common intracranial malignancy (12, 13), with an incidence of 20–40% based on autopsy data (14–16). The median survival generally ranges from 2 to 14 months, depending on prognostic factors, primary diagnosis, and tumor burden (1, 6, 17, 18). However, as many patients continue to live beyond the historical time range, WBRT is increasingly reserved for palliation in patients with large intracranial disease burden. More focal neurosurgical and stereotactic approaches are often the treatment of choice for patients with few (generally one to three) brain metastases or patients expected to be long-term survivors. How do we capture these survivors and guide them to the appropriate therapy?

In order to guide medical decision-making for patients with varied presentations and treatment options, several prognostic indices specifically addressing patients with brain metastases have been published in the literature. The most extensively utilized and validated prognostic system is the RTOG RPA, followed by the graded prognostic assessment (GPA) and diagnosis-specific graded prognostic assessment (DS-GPA) (1, 6, 7). However, significant heterogeneity in terms of patient populations, treatment approaches, patient factors assessed, statistical approaches, and number of prognostic groups has been observed between the various prognostic indices (19), demonstrating that such indices require ongoing improvement and optimization. The information obtained from the history and physical exam stands as the foundation for most of these indices as well as the clinician’s assessment of the patient’s cognition and prognosis. What information obtained from it, if any, is important for improving these indices?

In the RTOG RPA scoring system, univariate analysis of all covariates included neurological signs and symptoms as well as neurological function; however, these factors did not emerge as prognostic indicators in the overall analysis (1). Importantly, this index was based on patients undergoing conventionally fractionated whole brain radiotherapy (with one trial including a conventionally fractionated boost to GTV). In an attempt to diminish subjectivity and thereby improve reproducibility in these indices, the GPA and DS-GPA – which did include patients treated with radiosurgery – eliminated neurological function and physical exam findings in the regression tree analysis and only included easily quantifiable characteristics, such as age, KPS, number of CNS metastases, presence or absence of extracranial metastases, and primary diagnosis (6, 7). Ultimately, little data exist on the utility of the presenting neurological symptoms and signs noted on physical exam as prognostic indicators in patients treated with radiosurgery for brain metastases.

Our study included data from a multicenter prospectively collected database of 294 patients from three institutions treated with radiosurgery for brain metastases. In the multivariate analysis of standard elements of patient history and physical findings, neurological deficits noted on physical exam were not predictive of OS. In concordance with the previously published prognostic indices, historical factors predictive of OS in our study were primary diagnosis, ECOG performance status, and presence of extracranial metastases. Also consistent with previous reports, the presence of renal and breast histology conferred a better prognosis when compared to NSCLC or melanoma in our regression analysis.

The median survival of our patients was 10.8 months, and our patients were observed to maintain prolonged freedom from local recurrence (median 41 months). Additionally, patients completed SRS with minimal acute and chronic toxicity. These excellent results demonstrate that despite the importance of performance status on determining overall prognosis, worse performance status *specifically due to neurologic deficits* should likely be initially overlooked. Radiosurgery should continue to be offered to patients with intracranial metastases, especially if a patient’s performance status was normal prior to and compromised after presentation with a brain metastasis. The optimal treatment decision should be determined in a multidisciplinary setting where all treatment options are considered for each individual patient in the context of his or her presenting signs, symptoms, performance status, previous treatments, current disease status, and available options (20).

The ideal treatment paradigm for patients with brain metastases also continues to be optimized as new studies delineate the role of radiosurgery alone as initial therapy. Historically, the optimal treatment strategy for this patient population has varied significantly between treating centers and has been primarily based on the treating physician and patient preference. Recent randomized clinical trials have established the utility of radiosurgery alone without WBRT for patients with few (one to four) brain metastases (21–23). A recently published individual patient data meta-analysis of these three trials demonstrated an improvement in OS without an increased risk of distant brain failure for patients <50 years of age treated with radiosurgery alone (24). Similarly, recent results from the RTOG 0617/Alliance (NCCTG N0574) show that in patients with one to three brain metastases, when compared to WBRT plus radiosurgery, radiosurgery alone confers similar OS without a decline in cognitive function (25). The conclusion from these prospectively conducted studies is that for patients with low intracranial disease burden, young age, good performance status, and close follow-up, radiosurgery alone may be an adequate initial treatment strategy. Further studies will further optimize the treatment decision-making process based on prognostic and predictive factors reported here and elsewhere.

### Study Limitations

This was a retrospective study, and as such, there may be an inherent selection bias. Despite the maximal means undertaken to reduce or eliminate the effect of bias, the authors acknowledge that it is impossible to fully safeguard a retrospective study from such sources of analytical bias. This study was multi-institutional, and as such, our cohort of patients was evaluated by multiple physicians who may determine performance status and neurological deficits differently from each other. However, this is not a unique limitation to our study because even single-institution reports include patients treated by multiple physicians.

Conceptually, this study aims to describe a new patient presenting as a consultation with new brain metastases and possible neurological deficits. However, not all patients present to practicing radiation oncologists and neurosurgeons with this scenario, and such examples include an unknown primary or a patient who has already received intracranial therapy multiple times. Furthermore, our analysis is broad, and it is possible that the significance of the PMH (comorbidities) varies for specific histologic subtypes of cancer. For example, in patients with hormone positive breast carcinoma, PMH may be a significant predictor of clinical decision making and outcomes.

## Conclusion

Neurological deficits noted at presentation are not associated with worse overall prognosis for patients with brain metastases undergoing radiosurgery. When encountering new patients with brain metastases, the most informative patient-related characteristics that determine prognosis remain performance status ­(independent of recent neurological compromise), primary diagnosis, and presence of extracranial metastases.

## Author Contributions

All authors listed, have made substantial, direct and intellectual contribution to the work, and approved it for publication.

## Conflict of Interest Statement

SC and BC serve as clinical consultants to Accuray Inc. The Department of Radiation Medicine at Georgetown University Hospital receives a grant from Accuray to support a research coordinator. The other authors declare that they have no competing interests.
